# King Edward's Fund and the London Voluntary Hospitals

**Published:** 1920-03-27

**Authors:** 


### The Hospital

The Workers' Newspaper of Administrative Medicine and Institutional Life, Administration, National Insurance and Health.

No. 1764, Vol. LXVII. SATURDAY, MARCH 27, 1920. PRICE TWOPENCE

### KING EDWARD'S FUND AND THE LONDON VOLUNTARY HOSPITALS.

On another page will be found the full text of a very important Press statement which has been issued this week by the King Edward VII. Hospital Fund for London. The statement is terse, accurate, optimistic—even confident—in tone, and above all is highly opportune. No praise can be too great for the wisdom and courage displayed by the managers of the Fund in making this frank and open public pronouncement; and the expert views thus authoritatively voiced will not fail to make a deep impression upon the minds and consciences of London's citizens. This sort of thing, if we may be allowed to say so, is one of the legitimate functions, one of the bounden duties, of a body which has such peculiar powers of gathering and analysing authentic information as those possessed by the Council of the King's Fund. A great question, the future of the voluntary system in London, is, and has for some months, been at stake; and to enable the public to form a judgment on the issue the facts of the case need to be fairly set forth by some body in which general confidence is reposed.

There is no need to cavil over the timing of the statement. It is true enough that the position of the hospitals has been critical for months, as these columns have borne witness over and over and over again. Some readers may have thought our reiteration tiresome, but now that the view of the crisis so long urged is officially endorsed by the King's Fund, it will be seen that insistence was necessary. Critical as the situation certainly was in the latter half of 1919, it is doubly so in 1920; and now is the right time to redouble every effort to meet the crisis and relieve the strain of it upon the hospitals. On the whole, therefore, it is probably for the best that the King's Fund should have postponed until now their verdict on the facts and their call to action. The form, too, of the statement is excellent. There is no panic, no straining after effect, just simple statements all the more convincing because they are unadorned by rhetoric. The main principles upon which the Fund acts in deciding on its available sum for each annual distribution are revealed; they are sound ones, as was to be expected. "The hospitals of London," says the statement, "have to be tided over a period of difficulty . . . . but there is no reason to fear that the present difficulties cannot, if sufficiently energetic steps are taken, be successfully surmounted." The whole crux of the matter, as has long been pointed out in The Hospital, is contained in the phrase "sufficiently energetic steps." What these steps should be, must be, there is no need to repeat in detail now; they have been described many times in this paper during the past twelve months. Broadly speaking, they mean the better organisation of support for the hospitals, on a wider basis; the conversion, in fact, of as many as possible of the ninety or ninety-five per cent. of the population who at present contribute nothing into regular supporters in accordance with their means. This is easier to demand than to carry out; but where there is a will there is a way, and it is only if the will is lacking that the way will fail to be discovered.

The present system, the King's Fund statement concludes, in London alone provided last year nearly £2,000,000 towards a total expenditure of less than £2,200,000. The difference between expenditure and income may well be greater this year. The difficulties will not be solved without a resolute effort, but there is no reason to consider them insoluble. These are brave words, and words of wisdom. It is because the bravest course is usually also the wisest that we commend them especially to hospital committees and secretaries. Once they are driven well home to the individual men and women of London, all will be well. But no mere Press campaign can do this. Nor can the munificence of occasional generous donors who give or bequeath large sums to individual institutions or to the King Edward VII. Fund. Such deeds of charity by the very wealthy are, certainly, of great help; but they cannot replace the steady, regular, reliable flow of hundreds or thousands of small subscriptions. Personal service is what is needed in addition. The hospitals have been able to command it in the past, and, given intelligent organisation, there is little reason to suppose that they cannot rely upon a sufficiency of it in the future. What was a sufficiency a few years ago will not be a sufficiency now, that is all. The development of such service is what the voluntary hospitals must aim at, and it is sincerely to be trusted it will prove attainable.

